# Reframing human trafficking awareness campaigns in the United States: goals, audience, and content

**DOI:** 10.3389/fpubh.2023.1195005

**Published:** 2023-08-04

**Authors:** Elena Savoia, Rachael Piltch-Loeb, Daisy Muibu, Amy Leffler, Diana Hughes, Alberto Montrond

**Affiliations:** ^1^Harvard T.H. Chan School of Public Health, Harvard University, Boston, MA, United States; ^2^University of Alabama, Tuscaloosa, AL, United States; ^3^United States Department of Homeland Security, Washington, DC, United States

**Keywords:** human trafficking, awareness, prevention, labor trafficking, sex trafficking

## Abstract

**Introduction:**

Human trafficking (HT) awareness campaigns can educate the public and specific professional figures about this crime and ways to prevent it. However, there currently remains a gap in terms of how to best frame such campaigns without stigmatizing groups of individuals or portraying victims in unrealistic ways.

**Methods:**

We conducted four focus groups with 22 experts in HT to explore their perspectives and opinions on current challenges in the framing of HT awareness campaigns in the United States. Focus groups were conducted via Zoom and transcribed verbatim. Two independent reviewers analyzed the transcripts to identify themes using an inductive approach. The results of the focus groups analysis were used to structure the guiding questions of a brainstorming technique named Nominal Group Technique (NGT). Fifteen of 22 experts that participated in the focus groups joined the in-person NGT with the intent of generating ideas and achieving consensus on target audiences, goals, and content of human trafficking awareness campaigns. At the end of the NGT participants ranked priority for actions in the development of HT awareness efforts in the United States.

**Results:**

During the NGT the experts provided a number of recommendations to improve HT awareness and to empower victims to reach for help. They pointed to the need for: awareness efforts that describe HT on a spectrum of human abuse and exploitation; training for professional figures about trauma-informed care and communication; and efforts that empower trafficked victims to seek support. They also pointed to the need to develop awareness efforts tailored to local needs in close collaborations with the community-based organizations that can champion their dissemination and be the primary point of access for victims seeking help.

## Introduction

In the United States, human trafficking has been recognized as a federal crime for 23 years (1). While the exact count of trafficked victims in the country is unknown, in 2021 alone, 10,360 incidents of human trafficking were reported through the US National Human Trafficking Hotline (2). In addition, from 2015 to 2020, the US Department of Homeland Security (DHS) received 6,171 reports to their tip line regarding suspected human trafficking and child sexual exploitation (3). “In the fiscal year (FY) 2022, DHS opened 1,373 human trafficking investigations, an increase from 1,111 in FY 2021” (4). Moreover, “the Department of Justice (DOJ) opened 668 human trafficking investigations in FY 2022, an increase from 599 in FY 2021” (4). Among DOJ’s FY 2022 investigations, over 90% were related to sex-trafficking. “All U.S. states and territories have anti-trafficking criminal statutes. The federal government collects state, local, and tribal data on human trafficking investigations through the Uniform Crime Reporting Program (UCR Program), which includes data from participating jurisdictions of all 50 states. In 2021, participating jurisdictions reported 1,548 sex trafficking incidents and 294 labor trafficking incidents” (4). However, “not all agencies within all states are reporting human trafficking data to the UCR Program” and there is “no formal mechanism for the federal government to systematically track prosecutions at the state, local, and tribal levels” (4). Consequently, the data provided are likely to seriously underestimate this phenomenon.

The United States’ federal definition of human trafficking, as amended (22 USC § 7,102) in the TVPA of 2000, includes both sex and labor trafficking. The former is defined as the recruitment, harboring, transportation, provision, obtaining, patronizing, or soliciting of a person for the purpose of a commercial sex act, which is induced by force, fraud, or coercion, or if the person induced to perform it has not attained 18 years of age. The latter involves the recruitment, harboring, transportation, provision, or obtaining of a person for labor or services, through the use of force, fraud, or coercion for the purpose of subjection to involuntary servitude, peonage, debt bondage, or slavery (1). With the passage of the TVPA, this long-standing crime became prosecutable at the federal level, and the federal government has followed the 3P paradigm—prosecution, protection, and prevention—to combat human trafficking (5).

The Office on Trafficking in Persons of the US Department of Health and Human Services Administration for Children and Families (ACF) recommends examining prevention efforts through two models, namely the “Three Levels of Prevention Model,” which looks at efforts that can be made during different stages of the trafficking process, and the “Socio-Ecological Model,” which outlines the different risk factors that may contribute to being victimized. The Three Levels of Prevention model divides prevention work into primary, secondary, and tertiary prevention as typically done for public health interventions. In the context of human trafficking, primary prevention aims to prevent trafficking from occurring. Using human trafficking victimization as an example, when focused on a primary prevention effort, the aim is to intervene before the exploitation occurs. In other words, the goal of primary prevention efforts is to reduce the vulnerability of potential victims through empowering the individual and strengthening the social and community ties that can serve as additional protective barriers (6–8). Examples of primary prevention interventions include but are not limited to: spreading awareness about various form of exploitation (i.e., labor, sexual), educating the public about the risk of online grooming, educating children about the characteristics of healthy relationships, reducing social vulnerabilities like homelessness (7). Secondary prevention is an immediate response to trafficking victimization after it has occurred (6). It involves engagement of first responders (e.g., EMTs, law enforcement), as well as the provision of basic services, including emergency and medical care addressing short-term needs (6). These efforts remove victims from potential danger and provide them with the resources they need to temporarily escape their trafficking situation. Such interventions imply that someone will recognize the situation of victimization, and a major portion of secondary prevention work focuses on training professionals to identify possible trafficking victims and situations. An example is tattoo recognition. Because traffickers may mark victims, identification of tattoos provides a useful method for screening victims, which complements history taking, especially when victims are unable to disclose that information (9). Finally, tertiary prevention aims to prevent the victimization from reoccurring (6). Interventions at this level involve long-term rehabilitative services such as therapeutic counseling, job training, safe housing for victims, and relocation of victims among other potential supportive services.

Human trafficking campaigns can support all prevention levels by educating the public and specific professional figures on the topic. For example, awareness campaigns can be directed toward: youth to educate them about the risk of exploitation (primary prevention); healthcare professionals or law enforcement officers to educate them about the indicators of human trafficking (secondary prevention); or potential victims to inform them about local resources and organizations that can support them in their journey out of the condition of exploitation they are experiencing (tertiary prevention). These are few examples that describe how human trafficking campaigns can be implemented to enhance prevention efforts depending on their goals and target audience. This is particularly important nowadays due to advocacy and educational efforts being adversely impacted by disinformation (10). Specific communications can focus on the risks and vulnerabilities to human trafficking, criminal components of the act of trafficking, as well as traffickers’ manipulative recruitment strategies. Effective awareness campaigns should be able to aid in prevention and protection efforts (11). However, a gap exists in terms of how to best frame these types of awareness efforts to not only change knowledge, but also behaviors and attitudes about trafficking without stigmatizing groups of individuals or portraying victims in unrealistic ways (12).

In order to fill this gap, the current study aimed to gather information from human trafficking experts to better understand how to correctly frame such campaigns and more specifically to: (1) Identify challenges and areas for improvement in the development of human trafficking awareness efforts in the United States, and (2) Achieve consensus on priority areas for action in terms of goals, audiences and content of such campaigns. It is through such collaboration and connecting research to experts in the field, that more effective and meaningful awareness campaigns can be developed. The broader implication of such connections and collaboration between stakeholders is hopefully more informed approaches to HT awareness efforts, with more realistic and thoughtful content and goals.

## Methods

This study was based on the use of two methodological techniques: focus groups and nominal group technique (NGT). We first conducted a series of focus groups to explore human trafficking experts’ perspectives and opinions on current challenges in the framing of human trafficking awareness campaigns in the United States and opportunities for improvement. Following this exploratory phase we invited the human trafficking experts to an in-person meeting and implemented an NGT to generate ideas and achieve consensus on target audiences, goals, and content of such campaigns as it relates to primary and secondary prevention efforts. The study protocols for the focus groups and NGT were approved by the Institutional Review Board of the Harvard Longwood Medical Area.

### Focus groups

We invited 24 experts to participate in the study of which 22 replied. Four focus groups were convened between April 7 and 14, 2022 via Zoom, dividing the participants into groups of similar sizes: 8, 5, and two groups of 4, respectively. Participants were divided into groups based on their availability, while trying to balance the size of the groups to the extent possible.

Participants were selected from an initial list of non-governmental organizations and survivors working in the anti-trafficking field, as well as authors of reports and literature focused on trafficking. The list of participants was subsequently expanded through snowball referrals. The 22 experts represented various professional figures working in human trafficking prevention (each expert could represent more than one role), including survivors of human trafficking (*n* = 3), victim service providers (*n* = 16), academia (*n* = 3), and consultants and advocates (*n* = 3). A moderator’s guide was developed to stimulate the focus groups’ discussion around three guiding questions: (i) what should the goals of human trafficking awareness campaigns be?; (ii) whom should the target audience for these campaigns be?; (iii) and, what should the content of an effective awareness campaign include? The questions were inspired by existing guiding documents in health communication and social marketing (13, 14). The participants were presented with the guiding questions via email before the session commenced. A facilitator presented each question to the group to solicit opinions and further discussion. Individuals interacted via speaking or entering their comments into the Zoom chat function. A second facilitator moderated the questions and comments shared through the chat room. The focus groups were video recorded and transcribed. Participants consented to be interviewed and recorded.

#### Thematic analysis

Two analysts independently analyzed the transcripts and took notes on major themes emerging from the discussions using an inductive approach. The analysts adopted an inductive coding approach to derive the codes from the data without having a preconceived notion of what the codes should be but allow the themes to emerge from the raw data itself. This approach was well suited to meet the exploratory intent of this phase of the research during which we wanted the experts to come up with ideas and concepts. The two analysts met to compare their notes during two debriefing sessions until consensus was reached on the main issues (themes) discussed by the participants and the selection of the most appropriate and relevant quotes to include in the summary of results. Subsequently, the results were shared and discussed with the focus groups lead facilitator for accuracy and interpretation. The themes emerged from this analysis were subsequently used to structure the guiding questions for the NGT.

### Nominal group technique

The nominal group technique (NGT) took place between on May 20th 2022 in Boston, with the goal of further validating the results of the focus groups and moving from an exploratory phase into a consensus process to provide recommendations for the future development of campaign efforts. Participants were selected from the 22 participating in the focus groups. The 15 experts represented various professional figures working in human trafficking prevention, including the following non-mutually exclusive categories: survivors of human trafficking (*n* = 3), victim service providers (*n* = 11), academic experts (*n* = 3), and advocates (*n* = 7). All participants had over 10 years of experience serving victims of human trafficking and played leadership roles in advising government agencies in prevention efforts.

An NGT can be defined as a structured meeting that provides a structured procedure for obtaining qualitative information from target groups who are most closely associated with the problem area (15). It is primarily useful at the preliminary phase of a project, initiative, or study—or when a diverse group of stakeholders are brought together—as it offers a structured way both to generate a list of ideas relevant to the topic at hand and to quantify the group’s assessment of the ideas (via a ranking process) (16). NGT has largely been used in the fields of education and public health. Table 1 describes the NGT process. The purpose of the NGT meeting was to have participants achieve consensus as to the best way to develop and implement human trafficking awareness campaigns in the United States. Fifteen experts participated in the technique. The NGT designed for this meeting included an introduction to definitions of primary and secondary prevention efforts followed by a two-part brainstorming process: the first part was focused on the use of awareness campaigns for primary prevention efforts, and the second focused on the use of the campaigns for secondary prevention purposes. The human trafficking experts were divided in two groups (8 and 7 individuals respectively) based on their experience in human trafficking prevention efforts: one group focused on youth sex trafficking and the other one on adult trafficking (including labor and sex). Each group, guided by a trained facilitator and a note taker, implemented the technique to identify: (1) target audience, (2) goals and expected outcomes, and (3) content of the campaigns. At the end of the NGT session, the research team aggregated the notes, which derived from each group discussion and ranking. The results were reported back to the participants to clarify concepts and aggregate their ideas into major themes.

**Table 1:** Four Session NGT process (17).

Session goal	Description
Generating ideas	The moderator presents the question or problem to the group and directs everyone to write ideas in brief phrases or statements and to work silently and independently. Each person silently generates ideas and writes them down.
Recording ideas	Group members engage in a round-robin feedback session to concisely record each idea (without debate at this point). The moderator writes an idea from a group member on a flip chart, whiteboard, or screen that is visible to the entire group, and proceeds to ask for another idea from the next group member, and so on. This step ends when all members’ ideas have been documented.
Discussing ideas	Each recorded idea is then discussed to determine clarity and importance. For each idea, the moderator asks, “Are there any questions or comments group members would like to make about the item?” The moderator asks the group if there are any comments or questions for each idea that is recorded.
Voting on ideas—consensus	Individuals vote to prioritize the ideas. The votes are tallied to identify the ideas that are rated highest by the group as a whole.

## Results

In the sections below we first describe the results of the focus groups, followed by the results of the NGT.

### Focus groups results

See Table 2 for a list summary of the main challenges in the design and implementation of human trafficking campaigns identified by the focus groups’ participants identified.

**Table 2:** Challenges identified by focus group experts.

Challenge identified	Description
Evidence of impact	Lack of evidence on the effectiveness of human trafficking awareness campaigns.
Target audience	Lay people, with no professional training, or that only occasionally interact with victims, face difficulty recognizing instances of human trafficking. Lack of tailored training for those most likely to encounter at-risk or victims of human trafficking. Lack of engagement with victims and grassroot organizations in awareness and educational processes.
Content and goals	Difficulty in providing a realistic picture of the exploitation that human trafficking victims experience. Reluctance from community-based organizations in using awareness material produced by government agencies. Outdated foreign language materials.
Systemic issues	Unprepared to support victims once they are identified. Systematic issues that increase risk of labor trafficking.

The analysis of the four focus group transcripts led to the identification of four major themes: (1) Evidence of impact, (2) Target audience, (3) Content and goals, and (4) Systemic issues. Themes emerged organically with consistencies across groups, we have not identified opposing views or disagreement between participants or between groups. Each theme is described in detail below.

#### Evidence of impact

Focus group participants talked about the lack of evidence on the effectiveness of human trafficking awareness campaigns. As one participant working with victims of human trafficking and with past experience in implementing public awareness campaigns said, “*we have never seen evidence that says that these campaigns are effective*,” and they all shared concerns on the potential negative consequences of such campaigns, in particular, the deportation of undocumented immigrants and arrest of sex workers, or as a survivor described, “*the continued criminalization of individuals that are just merely trying to survive*.” The human trafficking experts underlined the importance of testing campaign materials before committing financial resources, as it is crucial to study both intended and unintended impacts of such efforts. Participants expanded on this issue, mentioning that there is a need for more evidence-based practices in the human trafficking prevention field and investments in researching what works. They also highlighted the need to gather data to better understand the extent of this crime, its prevalence, and characteristics of affected individuals so that awareness efforts can be better targeted and specialized services provided.

#### Target audience

Participants pointed to the unrealistic expectation that a lay person, with no professional training on the matter, may be able to make such a distinction during a five minute interaction with a potential victim. The experts remarked that only people who have received a significant amount of training are able to recognize an instance of human trafficking.

The participants said that the most effective way to raise awareness is to design campaigns that are directed to the victims themselves, in the words of one participant working for a hotline said, “*equipping people with knowing their rights*,” rather than directing efforts to people that may occasionally interact with them hoping they will recognize them as a potential victim. Raising awareness in victims about their rights is relatively simple. For example, in regards to labor rights, education should focus on minimum wage, safety of working conditions, and other important rights of working within the United States, but current campaigns lack this approach. They also emphasized that current efforts underestimate the ability of victims to react to their situation, as a survivor said: “*They have strength. They are the most resilient people we know. And what they lack is information and access to resources*.” The assumption that victims are unable to take action is undermining prevention efforts; as noted by a survivor: “*And what we need to do is stop with that assumption and focus on the assumption that trafficking survivors need information and access to resources only. We do not need people to rescue them. We do not need to report on them*.” Most victims, they continued, know they are in a situation of abuse, sometimes they do not know the abuse is illegal.

They discussed the importance of developing specialized training, rather than awareness efforts, targeting specific professional figures such as social workers, healthcare professionals, teachers, and attorneys (i.e., immigration attorneys) which may have opportunities and ways to interact with the victims. They emphasized that training should focus on how to ask questions to a potential victim and establish rapport in a way that is considerate of the ongoing trauma of the individual. They also expressed opinions on the importance of tailoring the training to the needs, circumstances, and culture of specific communities; as one participant with experience at the community level said, “…*tailor the message to the kind of exploitation the community might be facing*.” They remarked this is particularly important when a campaign is directed to communities where cultural, religious, and traditional belief systems may become an impediment to disclosing the situation. They pointed to the current lack of efforts that tailor the training to individuals who are most likely to encounter individuals at-risk or victims of human trafficking. They clarified that these groups are people that work for community-based organizations with high-risk populations (i.e., local healthcare centers providing care to immigrants, outreach workers, LGBTQ centers, runaway and homeless youth centers) as an expert working with immigrants referring to these categories of workers said “*those that have built trust with those communities…[those] that are more likely to be able to educate them to identify themselves where, when they are experiencing abuse and exploitation, and how to access services and support*.” As a focus group participant working with victims working in the agricultural industry stated, this would be more effective than “*educating folks (*i.e.*, law enforcement officers) that trafficking victims do not trust and hope that they’ll somehow disclose that they are a victim in a five-minute interaction when they are being triggered*.” The HT experts also emphasized that the training should include not only information on how to recognize instances of human trafficking, but also what services and resources victims may need. Focus group participants overall complained that there is currently a lack of engagement of grassroot organizations in awareness and educational processes despite the recognition that they are the best vehicle to access and provide support to the victims.

#### Content, source, and goals

Focus group participants highlighted the value of engaging survivors in the design of the campaigns to have them think about their life experience, the times when they could have reached for assistance, the places they went on a regular basis when they were victims, where and when they were alone, and when they were eventually in a position to write down a helpline number or ask for help. The participants expressed concerns about the fact that human trafficking cannot be comprehensively depicted in a short video or poster and that the result of this oversimplification leads to stereotyping, racism, sexism, and ultimately harm to the victims. They also talked about the fact that awareness campaigns do not do a good job in teaching about the distinction between adult sex trafficking and prostitution, as well as the nuances and difficulty of differentiating a consensual sex act from a non-consensual one. They acknowledged the difficulty in providing a realistic and comprehensive picture of the wide range of exploitation that human trafficking victims may experience. They argued that journalists and the movie entertainment industry are contributing to the wrong depiction of what trafficking looks like, focusing on the sensationalism of this phenomenon, and using an imaginary that evokes fear and portrays victims in a dehumanized manner. As a participant currently working with victims of labor trafficking described: “*(HT) is complicated and nuanced, and the nuance is always stripped out. So the campaigns are always reduced to soundbites or like a phrase or a tagline or something, but trafficking looks different for every single person*.” The participants continued to talk about images that invoke and reinforce racial bias and stereotypes in current awareness efforts. This can be corrected, as a participant working in advocacy efforts suggested, “…*by broadening the representation of those exposed to human trafficking…which is anybody*” and making sure that campaigns’ materials are inclusive of gender identities, race, and ethnic differences. They mentioned that it would be helpful to leverage digital technology to allow for the awareness material to be targeted to different communities and informational needs when individuals search for information in the online space.

When campaigns are designed to educate the greatest number of individuals in the US population, rather than focusing on individuals who due to their specific professional role (i.e., law enforcement officers, or healthcare professionals) may be in a position to come across and detect instances of HT, the participants suggested that the goal should be to raise sincere concerns about this crime so as to, as a survivor said, “… help shift not just the minds, but the hearts of the general public on this topic.” The HT experts also talked about reluctance from community-based organizations in using awareness material produced by government agencies, especially when Immigration and Customs Enforcement (ICE) hosts one of the hotlines and is provided as a source to report suspected cases. The contradictory role played by immigration officers (employed by ICE) and homeland security agencies (such as DHS) was discussed as being counterproductive, because victim protection and enforcement functions are in contradiction with each other. As a solution, they proposed having community-based organizations listed as a primary source to contact for awareness materials. In this regard, they also discussed the need for having better trained professionals answering the helplines.

The experts also discussed the need to develop materials in languages other than English and that most materials being used today in foreign languages are outdated. Referring to these outdated materials, a participant with experience in providing legal services to victims of human trafficking reported, “*it is not reflective of what we see on the street*.”

#### Systemic issues

Focus group participants pointed out the challenge of having a system unprepared to support the victims once they are identified; as a participant working with victims of sexual exploitation said, “…*awareness is relatively useless unless there is a specific call to action*” and continued by saying that the success of an awareness campaign should be measured in terms of how much support the victims that have been identified through the campaign have received. They expressed concerns that response gaps related to the post-identification process undermine the credibility of awareness efforts. They also expressed concerns about a criminal legal system that is arresting victims rather than protecting them, and that sometimes this is due to law enforcement officers not listening to the victims’ story, as a participant working with immigrant victims of labor trafficking said: “*They (law enforcement officers) do not believe our clients, they do not investigate labor trafficking.*” The focus groups’ participants argued trafficking cases are not prosecuted as needed, especially labor trafficking cases, and that victims, when identified, do not have access to basic resources such as housing, clothes, and food. The group continued by highlighting the need to develop policies that also protect those who are reporting the victims.

Focus group participants also stressed systemic issues that increase the risk of labor trafficking, such as the temporary visa system for workers in the agriculture and domestic labor market. As one participant working with victims of labor trafficking argued, “*We’ve created policies in which people are put into positions of abuse and exploitation through our refusal to enact worker protections*” and emphasized the need for greater oversight on this issue. The HT experts acknowledged the need to ensure that awareness about servitude and other types of labor trafficking is enhanced, as most efforts to date have focused on sex trafficking. Acknowledging the systemic issues, they continued, is important to frame awareness and training efforts correctly. As a participant working to prevent human trafficking emphasized: “…*if we talk about the big systemic issues [it] can also inform how a campaign is done…[by] acknowledging the systemic factors that bring someone to being in the [HT] situation* versus *saying it was a choice*.”

### Nominal group technique results

As a result of the NGT the HT experts achieved consensus on priority areas for action in the design of future HT awareness campaigns. The consensus process addressed the design of campaigns for both primary and secondary prevention efforts related to youth sex trafficking and adult labor trafficking. The actions listed by the HT experts can be used by campaign developers to prioritize their goals, the audience to be targeted and the specific content and message to be included in the campaign material. Below we provide a description of the results based on how the two groups of experts were divided: (1) HT experts in sex trafficking prevention efforts; and (2) HT experts on labor trafficking prevention efforts. Table 3 includes a list of recommendations for practice derived from the NGT.

**Table 3:** Recommendations on what outcomes, target audience and content to prioritize in the design of youth sex trafficking and adult labor trafficking awareness campaigns.

**Youth sex trafficking awareness campaigns**					
**Primary prevention efforts**			**Secondary prevention efforts**		
**Outcomes**	**Audience**	**Content**	**Outcomes**	**Audience**	**Content**
Raise awareness about the multifaceted aspects of sex trafficking. Raise awareness about the characteristics of healthy romantic relationships. Raise public concern about human trafficking.	Child welfare workers. Teachers and school counselors. Middle and high school students.	Be locally relevant. Describe sex trafficking within a spectrum of abuse. Include messages that foster resilience to human trafficking.	Enhance victim self-identification. Improve access to services (i.e., housing). Improve the communication skills of those potentially interacting with victims.	Youth in the online space. Professionals working in the judicial system (i.e., lawyers, probation officers) Workers in youth congregational settings.	Describe HT within a spectrum of abuse and exploitation. Include messages of on legal rights. Include specific indicators of human trafficking.
Adult labor and sex trafficking awareness campaigns					
Primary prevention efforts			Secondary prevention efforts		
Outcomes	Audience	Content	Outcomes	Audience	Content
Raise awareness about abusive or exploitative relationships. Raise awareness of environmental factors that can contribute to trafficking (i.e., labor settings). Raise awareness about reporting mechanisms and alternatives to reporting.	Vulnerable workers (e.g., temporary visa holders, sex workers). Workers who regularly go to people’s homes (e.g., postal workers). People summoned for jury duty.	Explanation of what trafficking is and is not. Information from real lived experiences. Information on individuals’ legal rights.	Raise awareness about access to resources and services. Improve trauma-informed service delivery skills. Enhance government leadership buy-in to promote employees’ education on HT.	First responders. Government employees interacting with vulnerable individuals (e.g., criminal justice workers). Providers engaged in long-term client/support relationships with potential victims (e.g., primary care physicians).	Include information on practical steps to respond to specific situations. Operationalize the concept of trauma-informed care. List resources and services that may be locally available.

#### Youth sex trafficking awareness campaigns

The NGT group focused on youth sex trafficking awareness campaigns achieved consensus on key actions to be taken to design campaigns for primary prevention (i.e., educational efforts directed to raise awareness about the risk and protective factors associated with human trafficking) and secondary prevention efforts (i.e., educational efforts directed to identify victims of human trafficking).

##### Primary prevention

Below we summarize priority areas for action for primary prevention efforts designed to raise awareness and general knowledge about HT related to youth sex trafficking starting with the target audiences, expected outcomes, and content.

###### Audience

The NGT participants agreed that human trafficking awareness campaigns designed for primary prevention purposes should prioritize the following audiences: (1) child welfare workers; (2) teachers and school counselors; and (3) middle and high school students. The advisors discussed how trafficking can happen in any community, but also agreed that disadvantaged communities should be considered as a priority for action as they are more likely to be affected by this crime. Additional audiences discussed by the participants were after school program staff, foster parents, professionals working in gang intervention programs, faith-based organizations leaders and their staff, and pediatricians.

###### Outcomes

The NGT participants agreed that this type of primary prevention awareness campaigns should accomplish the following outcomes: (1) increase awareness about the fact that human trafficking is multifaceted and that a variety of circumstances may progress into victimization; (2) increase awareness about the characteristics of healthy relationships; and (3) raise public concern about this crime and activism for policy changes. An additional outcome discussed by the experts was increasing awareness that human trafficking can directly or indirectly affect any individual regardless of their social status.

###### Content

The NGT participants discussed the content of the awareness campaigns and agreed that such campaigns should include: (1) scenarios that are relevant to the local context and characteristics of the community targeted by the campaign; (2) a description of sex trafficking as being part of a larger spectrum and continuum of abuse; and (3) examples of ways to foster resilience and appropriately communicate with youth. Additional content discussed by the participants included: information that will reduce gender, racial, and ethnic bias around this issue; information that will increase empathy and reduce fear; material in multiple languages; and information that addresses sexual consent and sexual boundaries.

##### Secondary prevention

Below we summarize priority areas for action for secondary prevention efforts designed to increase identification of potential cases of HT, and results are provided to describe the audience of such campaigns, expected outcomes, and content.

###### Audience

The NGT participants agreed that human trafficking awareness campaigns designed for primary prevention purposes should prioritize the following audiences and settings: (1) youth in the digital space where they are more likely to engage in risky behaviors and be approached by potential abusers; (2) attorneys, probation officers, and court-appointment personnel assigned to youth cases; and (3) workers of congregate care settings for youth.

Additional audiences discussed by the advisors were: extra curricula staff that work with high-risk youth, personnel in community clinics, immigration officers, and immigrants service providers. The HT experts also highlighted that other audiences may depend on the characteristics of the local community targeted by the campaign.

###### Outcomes

The NGT participants agreed that human trafficking awareness campaigns designed for secondary prevention purposes should focus on the following outcomes: (1) improve victims’ self-identification; (2) enhance victims’ access to appropriate services; and (3) advance communication skills of those responding to a situation of abuse. Additional outcomes discussed by the advisors included: decrease the number of victims being detained and/or deported and decrease the number of victims being charged with crimes related to human trafficking.

###### Content

The NGT participants discussed the content of the awareness campaigns and agreed that such campaigns should include: (1) information on the fact that HT lies on a spectrum of human abuse and exploitation; (2) information of the type “know your rights;” and (3) condition-specific indicators. Regarding indicators the most frequently cited by the experts were an individual manifesting low interpersonal trust, fear of law enforcement, marked wariness, anxiety, signs of self-harm, memory problems, medical history of sexually transmitted diseases, frequent changes of residency, not being in possession of identification documents and criminal charges such as prostitution and drug charges frequently due to the individual not being recognized as a victim by law enforcement officers.

Additional content discussed by the advisors were: narratives that foster courage and motivate people in reporting abuse of rights (i.e., labor rights); how to respond to disclosures in a sensitive way; and recognize the role and position of influence (i.e., financial, psychological influence) that perpetrators may have in the life of the victim.

#### Adult labor and sex trafficking awareness campaigns

##### Primary prevention

Below we summarize priority areas for action for primary prevention efforts designed to raise awareness and general knowledge about HT in the adult population related to the audience of the campaign, expected outcomes, and content.

###### Audience

The NGT participants agreed that human trafficking awareness campaigns designed for primary prevention purposes should prioritize the following audiences: (1) vulnerable workers (e.g., temporary visa holders, workers in low wage jobs, individuals with food insecurity, sex workers); (2) types of workers who regularly go to or into people’s homes (e.g., postal workers, electric, gas, home repair, or cable workers); and (3) people summoned for jury duty. Additional audiences discussed by the experts were healthcare workers, social workers, and community leaders especially in minority or historically marginalized communities.

###### Outcomes

The NGT participants agreed that this type of primary prevention awareness campaigns should accomplish the following outcomes: (1) improve people’s knowledge about abusive or exploitative relationships and their legal rights; (2) shift knowledge of first responders on the context and environment that can contribute to trafficking (i.e., labor settings); and (3) improve people’s knowledge about reporting mechanisms and alternatives to reporting. An additional outcome discussed by the advisors was increasing people’s trust in available resources.

###### Content

The NGT participants discussed the content of the primary awareness campaigns and agreed that such campaigns should include: (1) explanation of what trafficking is and is not; (2) information from real lived experiences; and (3) information on individuals’ rights to empower them to act. Additional content discussed by the experts included: cultivating empathy for victims and individuals that may be vulnerable to exploitation, and inclusion of updated data on HT prevalence.

##### Secondary prevention

Below we summarize priority areas for action for secondary prevention efforts designed to increase identification of potential cases of HT, results are provided to describe the audience of such campaigns, expected outcomes, and content.

###### Audience

The NGT participants agreed that human trafficking awareness campaigns designed for secondary prevention purposes should prioritize the following audiences: (1) first responders (e.g., police, emergency department and emergency management workers, 911 operators); (2) government employees (e.g., criminal justice workers, child protection programs’ staff, department of labor inspectors, public health personnel); and (3) providers engaged in long-term client/support relationships with victims (e.g., therapists, primary care physicians).

###### Outcomes

The NGT participants agreed that human trafficking awareness campaigns designed for secondary prevention purposes should focus on the following outcomes: (1) improve the ability to identify resources and services outside of law enforcement to refer potential victims; (2) improve skills in trauma-informed service delivery including building patience, tolerance, empathy, and change in adapting practices when interacting with a potential victim; and (3) enhance leadership buy-in of government agencies to promote education of employees on victim interaction once a potential case is identified. An additional outcome discussed by the advisors was change in communication skills and practices with potential victims.

###### Content

The NGT participants discussed the content of the awareness campaigns and agreed that such campaigns should include: (1) material with detailed information on specific circumstances and providing practical steps to respond to specific situations; (2) include material that operationalize the concept of trauma-informed care; and (3) create campaigns that link to resources on additional materials and support that may be available locally. Additional issues discussed by the experts were related to the way the material is shared with the target audience, including the appropriateness of the messengers or trainers based on the target audience.

## Discussion

The results of this study emphasize the need to develop human trafficking awareness campaigns to prevent and respond to this phenomenon using a public health approach. The challenges and solutions described in this manuscript can be extrapolated to the majority of public health campaigns around sensitive topics, and issues at the nexus between public health and criminal justice.

The experts engaged in our study argued that raising awareness about the existence of human trafficking with the intent of identifying potential cases is not sufficient if victims cannot be helped and supported once identified. Applying a public health approach to this complex crime means to prioritize the needs of the victim and focus on their protection. The experts also underlined that awareness campaigns need to be part of a continuum of prevention efforts that address the social, economic, systemic, and cultural factors that influence this complex phenomenon.

In public health, there is a long tradition of using awareness campaigns to improve population health from which we can learn. In public health awareness campaigns are typically designed to change how people think about their health and raise awareness about the resources they can have access to so as to change unhealthy behaviors. Specific topics may be considered sensitive due to the political, ideological, legal or cultural implications associated with the issue being considered. Examples of topics that fall under this category include opioid use disorder, mental health, firearm possession among many others. Some of the results and recommendations derived from this analysis may be extrapolated to any public health campaign dealing with sensitive issues.

Any campaign, to be effective, needs to be tailored to the targeted population, the phrase “general public” reflects a construct that may be used in colloquial language but does not meet basic principles of social marketing. Social change starts with a thorough analysis of the informational and emotional needs of various segments of the population—who have the ability to take actions to control their life, behaviors and outcomes (13). As such the general public is made of segments of the populations that are defined based on their informational needs, perceptions, literacy level etc., as such the same message may be effective for a specific segment of the population and non-effective for another segment. Whenever possible messages should be tested prior to their implementation, and evaluation practices should be included in any campaign development effort and related budget. At a minimum, simple methods such as focus groups or interviews with the target audience should be included at the early stage of development.

In the case of human trafficking, audience segmentation is a complex task because these campaigns have a dual goal, help victims get the support they need to get out their situation of exploitation and stopping a crime. Often when there are too many stakeholders their messages get mixed, diluted, or there is inconsistency across sources. Ultimately this undermines the overall impact of prevention efforts. To ensure consistent messaging it is important that stakeholders working across sectors (i.e., law enforcement, public health, education, healthcare) and create opportunities to meet and have productive dialogues and coordinated plans to address this phenomenon.

Similar to other campaigns around sensitive public health topics (i.e., opioid use disorder), in order to achieve effective communication, it is necessary to center the goal of the campaign around the needs of the victim first (in this case, the trafficked person). The HT experts engaged in this study emphasized that this goal can be achieved by empowering the victim to recognize the situation of exploitation they are living in and informing them on how to seek support, and by training professionals that may come across cases of exploitation on how to create an environment of trust to allow the victim to speak up. Such training should focus on improving skills such as empathetic listening, patience, trust building, and interviewing by asking questions that are appropriate to the situation and trauma experienced by the victim.

The human trafficking experts engaged in this study expressed concerns about awareness campaigns being disseminated by law enforcement agencies because they believe that individuals with lack of trust in institutions and law enforcement would not feel comfortable in contacting them, especially when the victim is an immigrant or has a criminal history. This is consistent with campaign efforts in the public health space, where the producer of the campaign may be an obstacle and result in distortion or misrepresentation of the issue addressed in the campaign material. This issue has been evident in campaigns addressing alcohol and tobacco consumption developed and disseminated by the industry with stakes in sales, as well as nutrition and baby formula. The same is the case with pharmaceutical companies developing awareness campaigns about diseases for which they are promoting a treatment. The source of the campaign as well as the channel by which the campaign is being promoted are important considerations that may largely impact the success of a campaign. In public health communication science the role of trusted messengers has been studied and emphasized across a variety of studies ranging from homelessness, non-communicable diseases to vaccine hesitancy to name a few (18–20).

In public health, there is evidence about the effectiveness of mass media campaigns for behavior change for tobacco cessation, healthy sexual behaviors, and promotion of physical activity but there is little evidence of the impact of awareness efforts on other issues, for example in reducing the use of illicit drugs. Whether or not media campaigns can prompt calls to helpline has been studied in the case of smoking cessation (21). “Overall, the direction of effect looks” [promising,] . . . “with campaigns serving to prompt calls to quit-lines” (22), but due to the “variation in. . . the quality of [the] studies,. . . there is only moderate certainty in the strength of this finding” (21). Most studies have focused on the impact of awareness campaigns on knowledge about specific risks and intentions to change behaviors under the assumption that “behavior change happens incrementally or via changes in mediating variables such as changes in knowledge, attitudes, self-efficacy, and intentions” (21).

The HT experts engaged in this study also pointed to the necessity of leveraging digital technologies to reach diverse audiences so as to develop better targeted awareness efforts. Digital technologies are changing the way public health organizations reach their audience (23, 24). Unlike the public health campaigns of the past that primarily used television and print modes of communication, modern campaigns are increasingly using digital strategies to maximize their influence on health behaviors (25).

A major concern reiterated multiple times by the experts is the risk of generating stigma on affected populations based on the language and imagery included in awareness efforts. The HT experts acknowledged the need to focus prevention efforts in geographic areas and communities most at risk due to poverty and marginalization, but also expressed concerns about generating stigma around the identity of people living in such areas. As described by Burriss, stigma is a cruel form of social control that “*cut[s] a person off from the esteem and support of others…*” (26 as cited in 29). “This is of particular relevance in public health, where messaging campaigns are often designed to reduce unhealthy behaviors. . . through social disapproval [and in some cases] outright shaming” (e.g., smoking) (26 as cited in 29). In tobacco control, we often say “There is no such thing as a ‘smoker,’ there are only people who smoke” (28 as cited in 29). “This framing intentionally creates space to decouple behavior from identity, so that unhealthy behavior (i.e., smoking) can be actively denormalized without perpetuating stigma against those who engage in it” (29). However, denormalization becomes problematic when the unhealthy behavior is strongly associated with an individual identity to the point of resulting in discrimination. For example, recent mpox-prevention messaging has focused on raising awareness of this disease and prioritizing vaccination clinics for gay men. A side effect of this strategy has been a stigmatization of this community. Similarly, efforts to decrease opioid misuse have caused stigmatization of all individuals who take opioids by labeling a broad swath of patients manifesting with chronic pain and presenting at the emergency room as drug seekers (29). In the case of human trafficking the challenge is even greater because awareness campaigns need to discern identity from behavior, but also describe behaviors within the complexity of the context and situation in which the exploitation develops, which is frequently due to systemic challenges such as poverty and marginalization. The negative consequences of campaign efforts causing stigmatization may be counterproductive to the point of reducing access to resources by those who most need them, exacerbating mistrust in institutions and in professional figures who are in the best position to provide support to the victims. To avoid the risk of stigmatization, it is important that awareness efforts educate the public on the victimization process rather than on the profile of an ideal victim that does not exist, it is important to educate the public on the systemic issues that lead to victimization. It is imperative to raise cultural awareness about the systemic legal, cultural, and socio-economic issues that lead to the phenomenon of human trafficking to avoid racial stereotyping and break the cycle of stigmatization.

As described by Vyncke B et al. in 2018 (31) while referring to previous literature “the way in which issues are perceived is not necessarily based on facts about an objective reality but is instead a mental construction of such reality created while interacting with others” (32 as cited in 31). “Stigma is a clear example of a social construction because” . . . [a specific] “attribute. . . is only considered deviant because society has defined it as such” (33 as cited in 31). As such, attributes may be stigmatizing an individual in certain societies, or historical periods, but can be considered normal in others. “A frame can be thought of as a narrative that focuses on specific” [elements] “of an issue and ignores others” (31). The literature shows that framing can influence the way the audience thinks about specific issues and acts upon the information received, including issues related to health and illnesses. Reframing an issue means to offer “the public a novel way of looking at” it offers alternative viewpoints (34 as cited in 31). The results of this analysis and recommendations provided by the HT experts engaged in this project point to the need of re-framing human trafficking awareness efforts to enhance prevention. Any reframing process starts with a listening phase during which those in charge of the communication listen to the experts bringing a different perspective. This manuscript aims to facilitate this listening phase. Following this listening phase, it is important that different types of stakeholders engaged in prevention efforts meet to further develop awareness efforts through iterative efforts where campaigns are developed, evaluated, and revised with constant feedback from the audience and experts on the topic. Reframing current human trafficking awareness campaigns will allow for the creation of products that are more effective and accepted by leaders, including survivors and service providers, within the anti-trafficking movement who are those best equipped to champion their dissemination.

## Conclusion

Through a series of focus groups and a consensus process this study highlighted the challenges and opportunities for improvement of current human trafficking awareness efforts in the United States. The experts engaged in this study discussed the need for awareness efforts (primary prevention efforts) to describe human trafficking on a spectrum of human abuse and exploitation and noted that sex and labor trafficking should not be addressed separately. The advisors also discussed how secondary prevention campaigns—designed to train professionals in detecting cases of human trafficking—should be targeted only to professionals that have the opportunity to interact with the potential victim for a prolonged period and focus on training about empathetic communication, trauma-informed care rather than the identification of signs of trafficking. The experts also pointed out the need to enhance awareness among trafficked victims to allow them to self-identify and seek support. Among other issues, the experts pointed to the need to develop awareness tailored to local needs in close collaborations with the community-based organizations that can champion their dissemination and be the primary point of access for victims seeking help. Finally, the experts highlighted the fact that the effectiveness of any type of societal awareness effort will always depend on the magnitude of the issue being addressed and political will to solve it from a system perspective, in the case of human trafficking not addressing the systemic issues linked to the crime would always undermine the effectiveness and credibility of awareness effort.

## Data availability statement

The raw data supporting the conclusions of this article will be made available by the authors, without undue reservation.

## Ethics statement

The studies involving human participants were reviewed and approved by the Harvard Longwood Medical Area Internal Review Board Boston. The patients/participants provided their written informed consent to participate in this study.

## Author contributions

ES conceived of the project and spearheaded the research design, data collection, and drafting of the manuscript. RP-L was responsible for running the nominal group technique. AM and DH coordinated and managed the focus groups. AM contributed to the discussion section of the manuscript. AL and DM drafted the introduction and provided feedback on the discussion section of the manuscript and the overall manuscript. DM reviewed entire article and edited the manuscript’s citations. All authors contributed to the article and approved the submitted version.

## Funding

This study was funded by the Department of Homeland Security Science & Technology Directorate, a project titled “Evaluation of the Blue Campaign”, award # 21STFRG00012-01-00. The content and the view expressed are only those of the authors.

## Conflict of interest

The authors declare that the research was conducted in the absence of any commercial or financial relationships that could be construed as a potential conflict of interest.

## Publisher’s note

All claims expressed in this article are solely those of the authors and do not necessarily represent those of their affiliated organizations, or those of the publisher, the editors and the reviewers. Any product that may be evaluated in this article, or claim that may be made by its manufacturer, is not guaranteed or endorsed by the publisher.
