# Erratum: Reframing human trafficking awareness campaigns in the United States: goals, audience, and content

**DOI:** 10.3389/fpubh.2023.1351506

**Published:** 2023-12-15

**Authors:** Frontiers Production Office

**Affiliations:** Frontiers Media SA, Lausanne, Switzerland

**Keywords:** human trafficking, awareness, prevention, labor trafficking, sex trafficking

Due to a production error, the following references were erroneously omitted from the web HTML version and XML file;

4. U.S. Department of State. *2023 Trafficking in Persons Report: United States*. Office to Monitor and Combat Trafficking in Persons (2023).

17. CDC. *Evaluation Brief: Gaining Consensus Among Stakeholders Through the Nominal Group Technique. Centers for Disease Control and Prevention* (2006). Available online at: https://www.cdc.gov/healthyyouth/evaluation/pdf/brief7.txt

The publisher apologizes for this mistake. The original article has been updated.

