# Corrigendum: Reframing human trafficking awareness campaigns in the United States: goals, audience, and content

**DOI:** 10.3389/fpubh.2024.1416730

**Published:** 2024-05-09

**Authors:** Elena Savoia, Rachael Piltch-Loeb, Daisy Muibu, Amy Leffler, Diana Hughes, Alberto Montrond

**Affiliations:** ^1^Harvard T.H. Chan School of Public Health, Harvard University, Boston, MA, United States; ^2^University of Alabama, Tuscaloosa, AL, United States; ^3^United States Department of Homeland Security, Washington, DC, United States

**Keywords:** human trafficking, awareness, prevention, labor trafficking, sex trafficking

In the published article, an author name was incorrectly written as “Alberto Montrod”. The correct spelling is “Alberto Montrond”.

In the published article, there was an error in the correspondence. The email address was incorrectly written as “dcmuibu@ua.edu”. The correct email address is “daisymuibu@gmail.com”.

In the published article citation number “4” for [US Department of State (2023): 2023 Trafficking in Persons Report: United States] was misplaced after “50 states.” It should have been placed after “tribal levels”. The corrected section of text appears below:

“All U.S. states and territories have anti-trafficking criminal statutes. The federal government collects state, local, and tribal data on human trafficking investigations through the Uniform Crime Reporting Program (UCR Program), which includes data from participating jurisdictions of all 50 states. In 2021, participating jurisdictions reported 1,548 sex trafficking incidents and 294 labor trafficking incidents. However, not all agencies within all states are reporting human trafficking data to the UCR Program and there is no formal mechanism for the federal government to systematically track prosecutions at the state, local, and tribal levels” (4).

In the published article the citation for [Stead M, Angus K, Langley T, Katikireddi SV, Hinds K, Hilton S, et al. Mass media to communicate public health messages in six health topic areas: a systematic review and other reviews of the evidence. Southampton): NIHR Journals Library (2019)] was misplaced after “this finding”. The citation has now been correctly placed with quotes and should read:

Most studies have focused on the impact of awareness campaigns on knowledge about specific risks and intentions to change behaviors under the assumption that “behavior change happens incrementally or via changes in mediating variables such as changes in knowledge, attitudes, self-efficacy, and intentions” (21).

In the published article, the reference [Krawiec RJ, McGuire K, McInerny J, Malik M. *U.S. The Future of Public Health Campaigns. Digital Strategies for Amplifying Influence and Effectiveness* (2021). Available online at https://www2.deloitte.com/us/en/insights/industry/public-sector/successful-digital-public-health-campaigns.html (accessed December 14, 2023).] was not cited in the article. The citation has now been inserted in **Discussion**, paragraph nine and should read:

“Unlike the public health campaigns of the past that primarily used television and print modes of communication, modern campaigns are increasingly using digital strategies to maximize their influence on health behaviors” (25).

In the published article, the reference for citation number 27 was incorrectly written as [BMJ. Word wars and tobacco control: choose the winner BMJ Blogs (2010) Available at: https://blogs.bmj.com/tc/2010/10/27/word-wars-and-tobacco-control-choose-thewinner/.Insert full reference].

It should be “BMJ. Word wars and tobacco control: choose the winner BMJ Blogs (2010) Available at: https://blogs.bmj.com/tc/2010/10/27/word-wars-and-tobacco-control-choose-thewinner cited in Moore MD, Ali S, Burnich-Line D, Gonzales W, Stanton MV. Stigma, opioids, and public health messaging: the need to disentangle behavior from identity. *Am J Public Health*. (2020) 110:807–10. doi: 10.2105/AJPH.2020.305628”.

In the published article, the reference for citation number 29 was incorrectly written as [Gorp BV. (2007). The constructionist approach to framing: Bringing culture back in. Journal of Communication, 57, 60–78.].

It should be [Gorp BV. (2007). The constructionist approach to framing: Bringing culture back in. Journal of Communication, 57, 60–78 cited in Vyncke B, van Gorp B. An experimental examination of the effectiveness of framing strategies to reduce mental health stigma. *J Health Commun*. (2018) 23:899–908. doi: 10.1080/10810730.2018.1538272].

The authors apologizes for these errors and state that this does not change the scientific conclusions of the article in any way. The original article has been updated.
