# Corrigendum: Reframing human trafficking awareness campaigns in the United States: goals, audience, and content

**DOI:** 10.3389/fpubh.2024.1486311

**Published:** 2024-10-10

**Authors:** Elena Savoia, Rachael Piltch-Loeb, Daisy Muibu, Amy Leffler, Diana Hughes, Alberto Montrond

**Affiliations:** ^1^Harvard T.H. Chan School of Public Health, Harvard University, Boston, MA, United States; ^2^University of Alabama, Tuscaloosa, AL, United States; ^3^United States Department of Homeland Security, Washington, DC, United States

**Keywords:** human trafficking, awareness, prevention, labor trafficking, sex trafficking

In the published article, quotations marks followed by a reference number to indicate direct quotes were missing in sections.

In the **Introduction**, paragraph one, the sentence previously stated:

“In the fiscal year (FY) 2022, DHS opened 1,373 human trafficking investigations, an increase from 1,111 in FY 2021. Moreover, the Department of Justice (DOJ) opened 668 human trafficking investigations in FY 2022, an increase from 599 in FY 2021.”

The corrected sentence, with reference (4) cited, appears below:

““In the fiscal year (FY) 2022, DHS opened 1,373 human trafficking investigations, an increase from 1,111 in FY 2021” (4). Moreover, “the Department of Justice (DOJ) opened 668 human trafficking investigations in FY 2022, an increase from 599 in FY 2021” (4).”

In the **Introduction**, paragraph one, the sentence previously stated:

“All U.S. states and territories have anti-trafficking criminal statutes. The federal government collects state, local, and tribal data on human trafficking investigations through the Uniform Crime Reporting Program (UCR Program), which includes data from participating jurisdictions of all 50 states. In 2021, participating jurisdictions reported 1,548 sex trafficking incidents and 294 labor trafficking incidents. However, not all agencies within all states are reporting human trafficking data to the UCR Program and there is no formal mechanism for the federal government to systematically track prosecutions at the state, local, and tribal levels (4).”

The corrected sentence, with reference (4) cited, appears below:

““All U.S. states and territories have anti-trafficking criminal statutes. The federal government collects state, local, and tribal data on human trafficking investigations through the Uniform Crime Reporting Program (UCR Program), which includes data from participating jurisdictions of all 50 states. In 2021, participating jurisdictions reported 1,548 sex trafficking incidents and 294 labor trafficking incidents” (4). However, “not all agencies within all states are reporting human trafficking data to the UCR Program” and there is “no formal mechanism for the federal government to systematically track prosecutions at the state, local, and tribal levels” (4).”

In the **Discussion**, paragraph four, the sentence previously stated:

“Effective social change starts with a thorough understanding of the informational and emotional needs of various segments of the population—who have ultimate control over their life, behaviors and outcomes.”

The corrected sentence, with reference (13) cited, appears below:

“Social change starts with a thorough analysis of the informational and emotional needs of various segments of the population—who have the ability to take actions to control their life, behaviors and outcomes (13).”

In the **Discussion**, paragraph eight, the sentence previously stated:

“Overall, the direction of effect looks promising, with campaigns serving to prompt calls to quit-lines (22), but due to the variation in the quality of the studies, there is only moderate certainty in the strength of this finding.”

The corrected sentence, with reference (21) cited, appears below:

““Overall, the direction of effect looks” [promising,] … “with campaigns serving to prompt calls to quit-lines” (22), but due to the “variation in…the quality of [the] studies,…there is only moderate certainty in the strength of this finding” (21).”

In the **Discussion**, paragraph ten, the sentence previously stated:

“This is of particular relevance in public health, where messaging campaigns are often designed to reduce unhealthy behaviors through social disapproval and in some cases outright shaming. (e.g., smoking) (27). In tobacco control, we often say “There is no such thing as a ‘smoker,' there are only people who smoke” (28). This framing intentionally creates space to decouple behavior from identity, so that unhealthy behavior (i.e., smoking) can be actively denormalized without perpetuating stigma against those who engage in it (28).”

The corrected sentence, with reference (29) cited, appears below:

““This is of particular relevance in public health, where messaging campaigns are often designed to reduce unhealthy behaviors…through social disapproval [and in some cases] outright shaming” (e.g., smoking) (26 as cited in 29). In tobacco control, we often say “There is no such thing as a ‘smoker,' there are only people who smoke” (28 as cited in 29). “This framing intentionally creates space to decouple behavior from identity, so that unhealthy behavior (i.e., smoking) can be actively denormalized without perpetuating stigma against those who engage in it” (29).”

In the **Discussion**, paragraph 11, the sentence previously stated:

“In society, the way in which issues are perceived is not necessarily based on facts about an objective reality but is instead a mental construction of such reality created while interacting with others (30). Stigma is a clear example of a social construction because a specific attribute is only considered deviant because society has defined it as such (31). As such, attributes may be stigmatizing an individual in certain societies, or historical periods, but can be considered normal in others. A frame can be thought of as a narrative that focuses on specific elements of an issue and ignores others (30). The literature shows that framing can influence the way the audience thinks about specific issues and acts upon the information received, including issues related to health and illnesses. Reframing an issue means to offer the public a novel way of looking at it offering alternative viewpoints (32).”

The corrected sentence, with references (31–34) cited, appears below:

“As described by Vyncke B et al. in 2018 (31) while referring to previous literature “the way in which issues are perceived is not necessarily based on facts about an objective reality but is instead a mental construction of such reality created while interacting with others” (32 as cited in 31). “Stigma is a clear example of a social construction because” … [a specific] “attribute…is only considered deviant because society has defined it as such” (33 as cited in 31). As such, attributes may be stigmatizing an individual in certain societies, or historical periods, but can be considered normal in others. “A frame can be thought of as a narrative that focuses on specific” [elements] “of an issue and ignores others” (31). The literature shows that framing can influence the way the audience thinks about specific issues and acts upon the information received, including issues related to health and illnesses. Reframing an issue means to offer “the public a novel way of looking at” it offers alternative viewpoints (34 as cited in 31).”

In the published article, the reference details for references 26 and 28–34 were incorrect. The correct details appear in the Reference list below and are updated in the original article.

The authors apologize for these errors and state that this does not change the scientific conclusions of the article in any way. The original article has been updated.
